# Parsing clinical text using the state-of-the-art deep learning based parsers: a systematic comparison

**DOI:** 10.1186/s12911-019-0783-2

**Published:** 2019-04-04

**Authors:** Yaoyun Zhang, Firat Tiryaki, Min Jiang, Hua Xu

**Affiliations:** School of Biomedical Informatics, The University of Texas Health Science Center at Houston, Houston, TX 77030 USA

## Abstract

**Background:**

A shareable repository of clinical notes is critical for advancing natural language processing (NLP) research, and therefore a goal of many NLP researchers is to create a shareable repository of clinical notes, that has breadth (from multiple institutions) as well as depth (as much individual data as possible).

**Methods:**

We aimed to assess the degree to which individuals would be willing to contribute their health data to such a repository. A compact e-survey probed willingness to share demographic and clinical data categories. Participants were faculty, staff, and students in two geographically diverse major medical centers (Utah and New York). Such a sample could be expected to respond like a typical potential participant from the general public who is given complete and fully informed consent about the pros and cons of participating in a research study.

**Results:**

2140 respondents completed the surveys. 56% of respondents were “somewhat/definitely willing” to share clinical data *with* identifiers, while 89% of respondents were “somewhat (17%) /definitely willing (72%)” to share *without* identifiers. Results were consistent across gender, age, and education, but there were some differences by geographical region. Individuals were most reluctant (50–74%) sharing mental health, substance abuse, and domestic violence data.

**Conclusions:**

We conclude that a substantial fraction of potential patient participants, once educated about risks and benefits, would be willing to donate de-identified clinical data to a shared research repository. A slight majority even would be willing to share absent de-identification, suggesting that perceptions about data misuse are not a major concern. Such a repository of clinical notes should be invaluable for clinical NLP research and advancement.

## Background

Parsing is a NLP task to assign syntactic structures to sentences according to grammar. Depending on the formation of syntactic structures, currently parsers could be categorized into two major types: the constituency parsers which are dependent on constituency grammars to distinguish between terminal (word) and non-terminal (e.g., phrase) nodes [1]; and the dependency parsers which generates simplified parse trees of only terminal nodes without considering the interior constituents [2]. Moreover, the shallow semantic relations between pairs of terminal nodes are labeled as dependency relations by the parsers. Since the constituency parse trees could also be converted into dependency parse trees, dependency parsers are gaining increasing attention. Many downstream NLP tasks, such as relation extraction [3–5] and machine translation [6], are highly relied on the dependencies between syntactic components. Therefore, dependency parsers are widely applided in multiple NLP applications including in the medical domain.

Generally, dependency parsing can be categorized into two approaches: transition-based and graph-based [2]. Transition-based parsers [7, 8] takes a sequence of actions to produce a parse tree. At each stage of the parsing process, a action is chosen based on the ranking scores of all possible actions. In contrast, graph-based parsers [9] consider parsing as a structure prediction problem and choose the correct tree based on the ranking scores of all possible trees. The development of syntactic parsing approaches has gone through several stages. Early symbolic parsing mainly used manually created deterministic grammars. Promoted by available annotated corpora such as the English Penn Treebank generated from Wall Street Journals, machine learning based approaches have been widely used in syntactic parsing [9–11]. Various machine learning approaches have been developed to generate the optimal parse tree based on the distributional statistics learned from the annotated Treebanks [12]. Commonly used syntactic parsing systems with good performance include the systems developed by Collins [13], Stanford parser [14], and Charniak et al. [15], etc.

However, there are several challenges faced by the conventional machine learning based approaches for syntactic parsing. First, current parsers usually use a large number of features including both lexicalized, context and combination features. This makes them suffer from the data sparsity problem, without sufficient annotated data to estimate accurate feature weights statistically. Moreover, conventional parsing systems are mainly built from manually designed feature templates, which is time consuming and highly dependent on domain expertise and experiences, meanwhile with limited coverage of linguistic patterns. Such approaches are not generalizable to new datasets from different domains [16].

## Related work

One potential solution to address the above challenges is the applications of deep learning based, or multi-layer neural networks based approaches. Recently, there are increasing research efforts on deep learning based dependency parsing, especially by using the LSTM (long short term memory) RNN (recurrent neural networks) [17]. This line of works is based on two assumptions: first, the low dimensional embeddings (distributional representation) features could alleviate the data sparsity problem; furthermore, the LSTM structure of each feature has the potential to represent their arbitrary feature combinations implicitly, reducing the explicit implementation of an explosive set of feature combinations [16]. Current works attempt to tailor the deep learning frameworks to dependency parsing from two aspects: (1) feature design: instead of using the previous templates of sparse, binary features, dense core features (i.e., words, part-of-speech taggings-POS and dependency labels) are encoded, concatenated and fed into non-linear classifiers such as multiple-layer perceptron [16–20]. (2) novel neural network architecture for feature encoding: Considering the design of neural network architectures is coupled with the feature set representation of parsers, stack-LSTMs [21] are used to describe the configurations (stack and buffer) of transition-based parsers, and hierarchical-LSTMs [22, 23] are used to encode the hierarchy of parse trees. Accordingly, the elements in the LSTMs are compositional representations of nodes in the parse trees. Le and Zuidema (2014) [22] and Zhu et al. (2015) [24] also employ rerankers, the input to the rerankers are encoded compositional representations capturing the structures around the node.

Currently, clinical NLP systems have been applied actively on narrative notes in EHR to extract important information facilitating various clinical and translational applications [5, 25]. Deep learning based methods have been applied to clinical NLP tasks such as concept recognition and relation extraction and obtained better performance in comparison to traditional machine learning methods [26]. Despite that syntactic parsers play a critical role in the NLP pipelines, existing dependency parsers with high-performance on the open text such as the Stanford Parser are usually directly applied in these system [27, 28]. Although some previous studies extended the traditional Stanford Parser using medical lexicons to tune it for clinical text [29], few efforts have been spent on investigating deep learning based dependency parsers for the clinical domain.

In our previous work, we systematically evaluated three state-of-the-art constituency parsers of the open domain including the Stanford parser and the Charniank parser and the berkly parser, and found that re-training the parsers using Treebanks annotated from clinical text improved the performance greatly [30]. Given the advantage of deep learning approaches for dependency parsers shown in general English text [16, 18, 19, 21–23], it’s timely to explore the performance of existing deep learning based dependency parsers, to set-up state-of-the-art performance and inform novel parsing approaches for clinical text.

## Objective

This study aims to investigate the performance of four open-sourced deep learning based dependency parsers on clinical text. Both transition-based parsers and graph-based parsers are evaluated, including Stanford parser [16], Bist-parser [20], dependency-tf parser [23] and jptdp parser [31]. The purposes of this study were three-fold: (1) to evaluate the default performance of existing state-of-the-art deep learning based dependency parsers on clinical text; (2) to examine the effect of clinical Treebanks for re-training general English parsers; and (3) to investigate the influence of pre-trained word embeddings from different large-scale unlabeled corpora on the performance of parsers on clinical text. The parsers are trained on the Penn Treebank in the original default settings. Moreover, a Treebank of progress notes [32] and the MiPACQ Treebank [33] are also used to retrain the parsers. In particular, the English Gigaword corpus of general text and MIMICIII, a corpus of clinical text, are used to train the word embedding models, respectively. To the best of our knowledge, this is the first comprehensive study that has investigated deep learning based dependency parsing of clinical text using multiple state-of-the-art dependency parsers and Treebanks from both the general English domain and the clinical domain.

## Methods

### The clinical treebank

In this study, we used two clinical Treebanks: 1) the MiPACQ Treebank described in Albright et al. [33] 2) the Progress Notes Treebank built in Fan et al. [32] After removing fragments and short sentences with less than 5 tokens, we used 10,661 sentences in the MiPACQ Treebank and 1025 sentences in the progress notes Treebank for experiments.

### Dependency-based syntactic representation

**Fig. 1:**
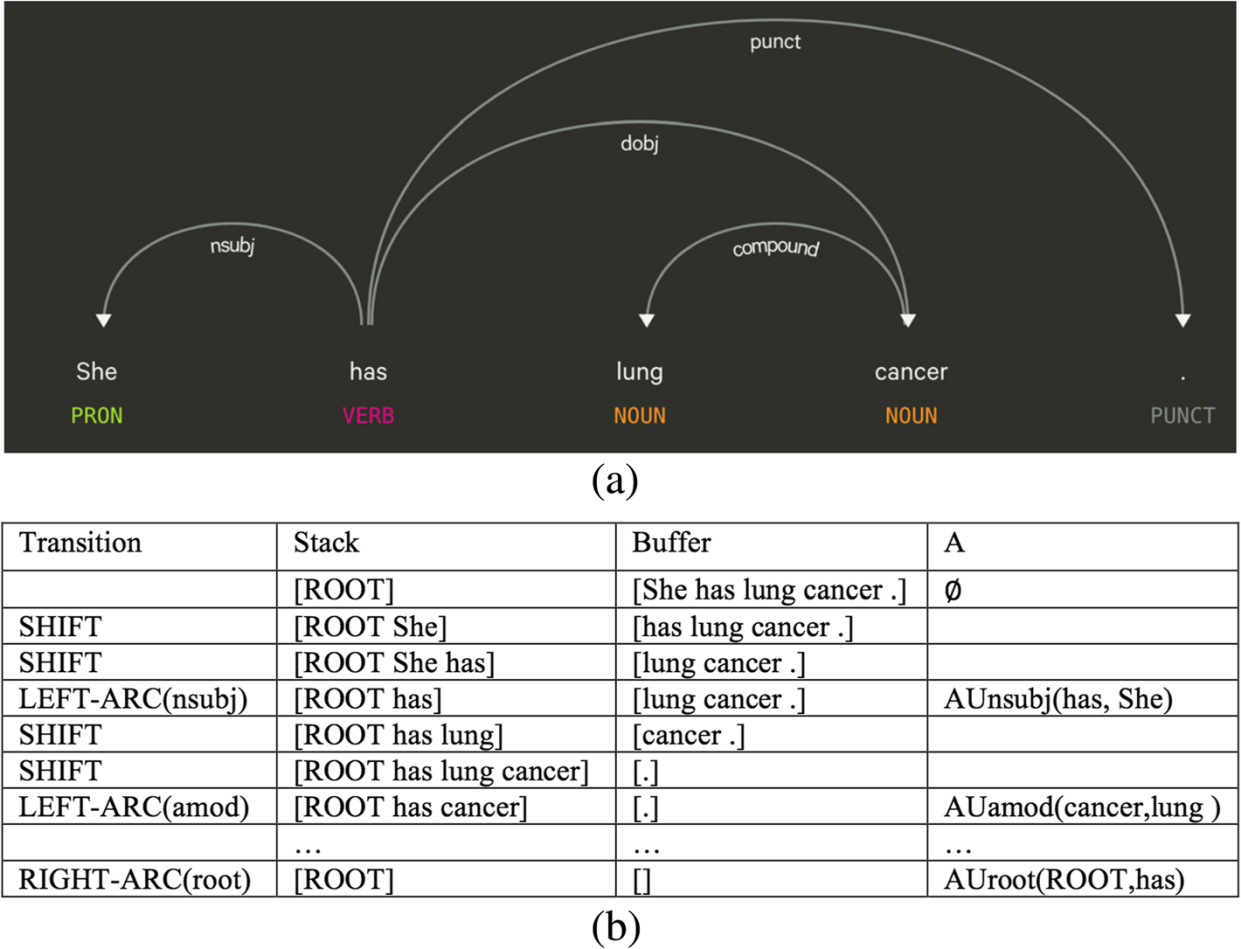
An example of a dependency parse tree and the transitions-based parsing process

A dependency-based syntactic tree represents a list of relations between head words and their modifier words [2]. Given a sentence with *k* words (*w*_*1*_,. .., *w*_*k*_), a dependency tree can be represented *k* relation triplets in the form of (*h*, *m*, *l*), where *h* and *m* stand for the indexs of a head word and a modifier word (0 ≤ *h* ≤ *k* and 1 ≤ *m* ≤ *k*), respectively. *h* and *m* represent two nodes in the tree and their pair forms an edge with label *l*, which is the index of a dependency relation in a set of *L* predefined dependency relations. An example of dependency tree of the sentence “She has lung cancer.” is illustrated in Fig. 1(a).

### Deep-learning based dependency parsers

#### Stanford parser

Chen and Manning (2014) [16] builds a greedy transition-based parser based on neural network architectures. For this, the arc-standard system is employed. This transition system consists of a set of configurations *c* = (*s*, *b*, *A*), where *s* stands for a stack, *b* stands for a buffer and *A* stands for a set of dependency arcs. Given a sentence *w*_*1*_, . . ., *w*_*k*_, the parsing process initiates a starting configuration, with *s* = [ROOT], *b* = [*w*_*1*_, . . ., *w*_*k*_], *A* = ∅. Given an intermedia configuration, where *s*_*i*_ is the *i*th top element on the stack and *b*_*i*_ is the *i*th element on the buffer, the system will choose from three types of transitions: LEFT-ARC(l) by adding an arc *s*_*1*_- > *s*_*2*_ with label *l* and remove *s*_*2*_ from the stack; RIGHT-ARC(*l*) by adding an arc *s*_*2*_- > *s*_*1*_ with label *l* and remove *s*_*1*_ from the stack; SHIFT where *b*_*1*_ if moved from the buffer to the stack. In each iteration, an optimal transition is automatically choosen, based on features extracted from the current configuration. The parsing process will undergo multiple iterations until a parse tree is formed. The process of transition-based parsing is illustrated in Fig. 1(b).

**Fig. 2:**
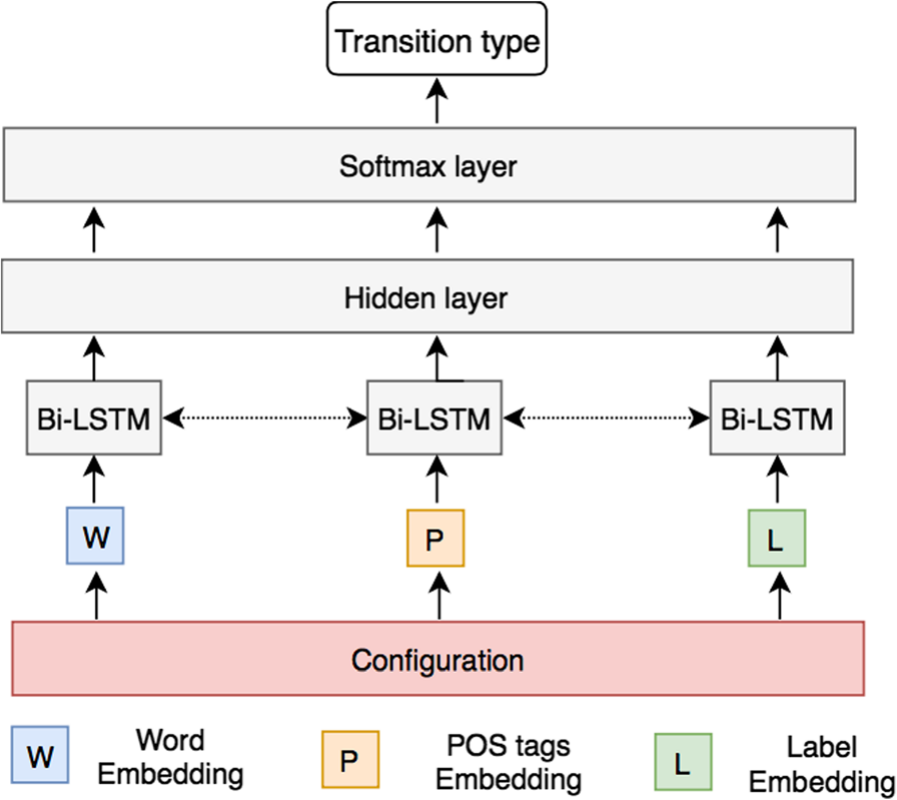
An example of the neural network architecture for the transitions-based parsing process

As illustrated in Fig. 2, a neural network with one hidden layer is used to classify the transition for each configuration. Dense low-dimensional features, or embeddings of a rich set of elements are used as features of the input layer. For example, eighteen elements of words (*x*) from the stack and buffer are used as features: (1) the top three words, the first and second leftmost/rightmost children and the leftmost of leftmost/rightmost of rightmost children of the top two words on the stack, and (2) the top three words on the buffer. Similarly, eighteen elements of POS tags (*x*^*t*^) and twelve elements of arc labels (*x*^*l*^) are used as features of the input layer, which encode the information of the current stack, buffer and arcs. The vectors are then concatenated and fed into a nonlinear classifier based on MLP. The function *h* = (*w*^*w*^*x*^*w*^ + *w*^*t*^*x*^*t*^ + *w*^*l*^*x*^*l*^ + *b*)^3^ in the hidden layer is expected to capture arbitrary feature combinations. Softmax probabilities is calculated based on a cross-entropy loss function in the output layer for transition classification.

#### Bist-parser

The neural network architecture used for the bist-parser [20] is similar with the Stanford parser. Different from the Stanford parser which uses concatenated embeddings of a rich set of elements (words, POS tagging and arc labels) as features, only the top 3 words (*S*_*2*_, *S*_*1*_ and *S*_*0*_) on the stack and the first word on the buffer (*b*_*0*_) are used and their feature vectors are concatenated to form the minimal feature function in the bist-parser. The feature vector of each word is a concatenation of embeddings of the word and its POS tag. Besides, a hinge loss function is defined based on each parsing configuration c:

$$ \mathit{\max}\left(0,1-\underset{t_o\in G}{\mathit{\max}} MLP\left(\varnothing (c)\right)\left[{t}_o\right]+\underset{t_p\in A\backslash G}{\mathit{\max}} MLP\left(\varnothing (c)\right)\left[{t}_p\right]\right) $$

Where *A* and *G* stand for the sets of possible transitions and correct transitions at the current configuration c.

#### Dependency-tf parser

Kiperwasser and Goldberg (2016b) [23] propose a tree encoding that naturally supports trees with arbitrary branching factors, making them particularly suitable for dependency trees. Tree encoder uses RNN as the building block: RNN is used to model the left and right sequences of modifiers, which are constructed recursively to form trees. Representation of parsing trees is constructed to in a greedy, bottom-up way based on the Easy First Transition System by Goldberg and Elhadad (2010) [34].

#### jPDTP parser

This work constructs a novel neural architecture [31] for **j**oint **P**OS **t**agging and **d**ependency **p**arsing (jPDTP). The parser uses biLSTM (bidirectional LSTM) [17] to learn shared latent features representing word tokens in input sentences. These shared features are then further used for POS tag prediction, which are also fed to a MLP with a hidden layer to decode dependency arcs and another MLP to predict relation types for labeling predicted arcs. Specifically, in order to improve the performance of POS tagging, a character-based embedding sequence of each word is generated and connected with the word embedding of each word *w*_*i*_. In addition, the indexing position of each word in a sentence is also used as a feature. Latent features based on shared biLSTM are used to represent POS tags, and cross-entropy target loss is used to predict POS tags. Dependency trees are formalized as directed graphs. The arc-factored parsing approach learns arc scores in graphs. The decoding algorithm will find the maximum spanning tree from these arc scores-the parse tree with the highest score in the graph:

$$ score(s)=\underset{\widehat{y}\in Y(s)}{argmax}{\sum}_{\left(h,m\right)\in \widehat{y}}{score}_{arc}\left(h,m\right) $$

where *Y (s)* is the set of all possible dependency trees for the input sentence *s*, and *score*_*arc*_(*h*, *m*) measures the arc score between the head word and the modifier word in *s*. The arc is scored by using MLP with a single node output layer (MLParc) on the BiLSTM feature vectors:

$$ {score}_{arc}\left(h,m\right)={MLP}_{arc}\left({v}_h\circ {v}_m\right) $$

where *v*_*h*_ and *v*_*m*_ are biLSTM-based shared feature vectors representing the *h*_*th*_ and *m*_*th*_ words in *s*, respectively. Then, the model calculates the margin based hinge loss by using loss-augmented reasoning to maximize the margin between the unlabeled gold parse tree and the highest scoring incorrect tree. Dependency types are predicted in a similar way. Another MLP is used on the biLSTM feature vector to predict the relationship type of the head-modifier arc.

### The parsing experiments

Three experiments were conducted for each parser as described below:

- Use default settings to evaluate parser performance: In this experiment, we directly applied four parsers to handle all POS tagged sentences in the treebanks. All parsers were used in default settings that have been trained on Penn Treebank. The parse trees generated by each parser were then compared with the gold standard Treebank and the performance of each parser was reported (see the Evaluation section).
- Retraining parsers using word embeddings of open text and clinical Treebanks: In order to assess whether retraining of clinical corpora can improve the performance of parsers, we conducted a 10-fold cross-validation evaluation for each parser. Cross-validation includes dividing the clinical corpus into 10 parts, training parsers on 9 parts, and testing the remaining parts each time. We repeated the same program 10 times, once for each part, and then merged the results of 10 parts to report the performance. The default word embeddings pre-trained from the AFP portion of the English Gigaword corpus were used for each parser. [35]
- Re-train parsers on the clinical Treebank using word embeddings of clinical text: To further evaluate the effects of using word embeddings features generated from clinical text, we employed the word embeddings pre-trained from the MIMICIII corpus [36] and conducted 10-fold cross validation evaluation for each parser.

### Evaluation

As mentioned earlier, for each parser, we conducted the above three experiments using 10-fold cross validation. For each test sentence, the parse tree generated by the parser was compared with the corresponding gold standard in the Treebank.

For each sentence, the following metrics commonly reported for dependency parsers are used:

Unlabeled attachment score (UAS) = (The number of arcs assigned correctly)/(The number of assigned arcs);

Labeled attachment score (LAS) = (The number of predicted dependencies where the arc and the label are assigned correctly)/(The number of predicted dependencies);

Label accuracy score (LS) = (The number of dependency labels assigned correctly)/(The number of predicted dependency labels).

## Results

Table 1 illustrates the experimental results on the MiPACQ Treebank. The Bist-parser achieved the optimal performance by using the default Penn TreeBank and word embeddings of Gigaword for training; while the jPTDP parser obtained the lowest performance of 77.59% UAS, 83.60% LA and 71.58% LAS. Re-training on the clinical Treebank improved the performance for all the four parsers. Applying word embeddings generated from Gigaword produced slightly better performance than word embeddings of MIMICIII for stanford parser and Bist-parser. Overall, the Bist-parser obtained the optimal performance of 90.72% UAS, 95.18% LS and 89.25% LAS, retrained on the MiPACQ TreeBank using word embeddings from Gigaword. In contrast, the largest improvement was obtained by using clinical data for the Jptdp parser. Retraining the parser increased the performance sharply (88.50% UAS, 92.36% LS and 85.53% LAS). Using the word embeddings from MIMICIII further improved the performance over Gigaword (88.95%UAS, 92.69% LS, 86.10% LAS).

**Table 1:** Performance of deep learning based dependency parsers on the MiPACQ corpus (%)

Parser	Corpus	Word embeddings	UAS	LS	LAS
Stanford parser	Penn TreeBank	Gigaword	80.62	89.09	77.59
	MiPACQ	Gigaword	90.49	94.95	89.00
	MiPACQ	MIMICIII	90.30	94.84	88.75
Bist-parser	Penn TreeBank	Gigaword	81.08	89.35	78.20
	MiPACQ	Gigaword	**90.72**	**95.18**	**89.25**
	MiPACQ	MIMICIII	90.62	95.16	89.16
Dependency-tf	Penn TreeBank	Gigaword	79.14		
	MiPACQ	Gigaword	88.65		
	MiPACQ	MIMICIII	88.80		
jPTDP-parser	Penn TreeBank	Gigaword	79.47	85.76	74.62
	MiPACQ	Gigaword	88.50	92.36	85.53
	MiPACQ	MIMICIII	88.94	92.69	86.10

Highest performance in terms of each evaluation criterion is highlighted in boldface

Table 2 illustrated the dependency parsing results obtained using the progress notes. The overall trend of the performance in different settings is similar with that of MiPACQ. However, the performance of the progress notes is much lower than the performance of MiPACQ. The highest performance was produced by using the Stanford parser, retrained on the progress notes (84.01% UAS, 90.16% LS, 80.72% LAS).

**Table 2:** Performance of deep learning based dependency parsers on the progress notes (%)

Parser	Corpus	Word embeddings	UAS	LS	LAS
Stanford parser	Penn TreeBank	Gigaword	75.76	84.23	71.21
	ProgressNotes	Gigaword	**84.01**	90.00	**80.72**
	ProgressNotes	MIMICIII	**84.01**	**90.16**	80.66
Bist-parser	Penn TreeBank	Gigaword	75.01	84.73	71.05
	ProgressNotes	Gigaword	82.26	89.69	78.94
	ProgressNotes	MIMICIII	81.78	89.31	78.42
Dependency-tf	Penn TreeBank	Gigaword	78.02		
	ProgressNotes	Gigaword	76.72		
	ProgressNotes	MIMICIII	77.09		
jPTDP-parser	Penn TreeBank	Gigaword	75.51	73.47	60.24
	ProgressNotes	Gigaword	77.59	83.60	71.58
	ProgressNotes	MIMICIII	79.35	85.10	73.61

Highest performance in terms of each evaluation criterion is highlighted in boldface

## Discussion

Dependency parsers are commonly used as one essential module in the pipelines of important clinical NLP tasks such as named entity recognition and relation extraction. A dependency parser of high-quality is critical to the final output of the clinical NLP system and related applications in the medical domain. Given that deep learning based syntactic parsers achieve the state-of-the-art performance on open text, it is timely for this study to compare and evaluate deep learning based dependency parsers on clinical text.

Our results showed that, compared with open text, the original parser achieves lower performance in clinical text. For example, on the MIPACQ corpus, Bist-parser showed significant decreases in both UAS and LAS (UAS: 93.2 to 81.08%, LAS: 91.2 to 78.20%). After retraining on clinical Treebanks, all parsers achieved better performance. This indicates that retraining on clinical TreeBanks is necessary for the development of high-performance dependency parsers for clinical texts. In addition, it proves that it is essential to build customized Treebanks for clinical texts.

To validate the advantage and necessity of using deep learning based approaches for dependency parsers, we further compared the published performance of dependency parsers built using conventional machine learning methods. Albright et al. [33] applied the Clear dependency parser to MiPACQ, which is a transition-based parser built using the Support Vector Machine (SVM). The reported performance of the parser trained on the Penn TreeBank is a 78.34% UAS and a 74.37% LAS [33], in contrast to a 80.62% UAS and a 77.59% LAS using the deep learning based Stanford parser trained on the same TreeBank. In addition, the reported performance of the parser trained on MiPACQ is a 85.72% UAS and a 83.63% LAS [33], in contrast to a 90.30% UAS and a 88.75% LAS using the deep learning based Stanford parser also trained on MiPACQ. The original dataset setting in the work of Albright et al. [33] was 85% for training, 5% for development and 10% for test, whereas we used 10-fold cv in this study. Despite the different experiment configurations, sharp improvements (2.32% ~ 5.12%) can be observed when using deep learning approaches for dependency parsers, especially when retrained using the MiPACQ data and word embeddings of MIMICIII.

It is noteworthy that the use of word embeddings of Gigaword and MIMICIIII has yielded comparable performance. In fact, word embeddings of Gigaword were used in two parsers that achieved the best performance, Bist-parser (88.95% UAS, 92.69% LS, 86.10% LAS) on MiPACQ and Stanford parser (84.01% UAS, 89/97)% LS, 80.72% LAS) on progress records. Despite that Gigaword is a corpus of open text, and MIMICIII is a corpus of clinical text. One potential reason is that the clinical text is a mixture of linguistic elements of general English and elements unique to the medical domain. Although both Gigaword and MIMICIII have domain gaps with MiPACQ and the progress notes, they still contribute to the task with generalizable syntactic and semantic characteristics learned in an unsupervised manner.

Among the four dependency parsers, Stanford parser and Bist parser are transition-based parsers, using the core features of each configuration to classify transition types. Dependency-tf parser is also a transition-based parser, which is characterized by encoding subgraphs of parsing trees recursively. JPTDP-parser is a graph-based parser that directly targets the best parsing tree. Interestingly, the Stanford parser and the Bist-parser obtained better performance than the other two parsers using graph-based features. This follows the same trend for transition-based and graph-based parsers when evaluated on the open text. [20] Besides, compared with graph-based parsers, transition-based parsers showed greater generalizability on different Treebanks. As an illustration, the Stanford parser got a 77.59 LAS using the default setting and a 88.75 LAS when retrained on MiPACQ. In contrast, the jPTDP-parser got a 71.58 LAS using the default setting and a 86.10 LAS when retrained on MiPACQ.

Our study has several limitations. Firstly, the focus of this study is mainly an initial evaluation of state-of-the-art deep learning based dependency parsers on clinical text. The coverage of current deep learning based dependency parsers is not comprehensive. Additional dependency parsers such as the SyntaxNet from Google [37] will be implemented and evaluated. Besides, only MiPACQ of colon cancer notes and progress notes from the i2b2 2010 challenge were used in the current study. The clinical TreeBanks will be enriched with other types of clinical notes such as discharge summaries and pathology notes by manual curation in our future work. Moreover, our previous study demonstrated that leveraging the dataset of the open domain such as the Penn TreeBank could improve the parser performance on clinical text. [30] Besides, the use of word embeddings of open text such as Gigaword produced performance comparable to that of using word embeddings of clinical notes as initial features of a dependent parser.

Therefore, we will investigate the combination of TreeBanks and word embeddings of external domains with clinical resources for any potential improvement to the deep learning based dependency parsers, in order to make full use of available resources and alleviate the heavy burden of clinical TreeBank curation.

## Conclusion

We conducted a formal evaluation to study the use of four state-of-the-art deep learning-based dependency parsers in the medical field. Our results showed that Bist-parser achieved the best performance when applied directly to clinical texts. In addition, retraining on the annotated clinical treebank significantly improves the performance of all parsers, indicating the need to create a large clinical treebanks. Moreover, experimental results demonstrated that word embeddings generated from open text could produce similar performance as word embeddings generated from clinical notes when used as the initial features of the parser. Therefore, more sophisticated use of corpora and word embeddings from external domains is worth studying for clinical parsing improvement.

## Abbreviations

biLSTM: Bidirectional LSTM
EHR: Electronic health record
jPDTP: **J**oint **P**OS **t**agging and **d**ependency **p**arsing
LSTM: Long short term memory
NLP: Natural Language Processing
RNN: Recurrent neural networks
